# *De novo* transcriptome in roots of switchgrass (*Panicum virgatum* L.) reveals gene expression dynamic and act network under alkaline salt stress

**DOI:** 10.1186/s12864-021-07368-w

**Published:** 2021-01-28

**Authors:** Pan Zhang, Tianqi Duo, Fengdan Wang, Xunzhong Zhang, Zouzhuan Yang, Guofu Hu

**Affiliations:** 1 College of Animal Science and Technology, Northeast Agricultural University, Harbin, 150030 China; 2 State Key Laboratory of Biocontrol, College of Life Science, Sun Yat-sen University, Guangzhou, 510006 China; 3 School of Plant and Environmental Sciences, Virginia Tech, Blacksburg, VA 24061 USA

**Keywords:** *Panicum virgatum*, Alkaline salt stress, ASTTI, Transcriptome, WGCNA

## Abstract

**Background:**

Soil salinization is a major limiting factor for crop cultivation. Switchgrass is a perennial rhizomatous bunchgrass that is considered an ideal plant for marginal lands, including sites with saline soil. Here we investigated the physiological responses and transcriptome changes in the roots of Alamo (alkaline-tolerant genotype) and AM-314/MS-155 (alkaline-sensitive genotype) under alkaline salt stress.

**Results:**

Alkaline salt stress significantly affected the membrane, osmotic adjustment and antioxidant systems in switchgrass roots, and the ASTTI values between Alamo and AM-314/MS-155 were divergent at different time points. A total of 108,319 unigenes were obtained after reassembly, including 73,636 unigenes in AM-314/MS-155 and 65,492 unigenes in Alamo. A total of 10,219 DEGs were identified, and the number of upregulated genes in Alamo was much greater than that in AM-314/MS-155 in both the early and late stages of alkaline salt stress. The DEGs in AM-314/MS-155 were mainly concentrated in the early stage, while Alamo showed greater advantages in the late stage. These DEGs were mainly enriched in plant-pathogen interactions, ubiquitin-mediated proteolysis and glycolysis/gluconeogenesis pathways. We characterized 1480 TF genes into 64 TF families, and the most abundant TF family was the C2H2 family, followed by the bZIP and bHLH families. A total of 1718 PKs were predicted, including CaMK, CDPK, MAPK and RLK. WGCNA revealed that the DEGs in the blue, brown, dark magenta and light steel blue 1 modules were associated with the physiological changes in roots of switchgrass under alkaline salt stress. The consistency between the qRT-PCR and RNA-Seq results confirmed the reliability of the RNA-seq sequencing data. A molecular regulatory network of the switchgrass response to alkaline salt stress was preliminarily constructed on the basis of transcriptional regulation and functional genes.

**Conclusions:**

Alkaline salt tolerance of switchgrass may be achieved by the regulation of ion homeostasis, transport proteins, detoxification, heat shock proteins, dehydration and sugar metabolism. These findings provide a comprehensive analysis of gene expression dynamic and act network induced by alkaline salt stress in two switchgrass genotypes and contribute to the understanding of the alkaline salt tolerance mechanism of switchgrass and the improvement of switchgrass germplasm.

**Supplementary Information:**

The online version contains supplementary material available at 10.1186/s12864-021-07368-w.

## Background

Soil salinization, a major limiting factor for crop cultivation, seriously restricts global agricultural production and ecological environment construction [1, 2]. Soil salinization may cause neutral salt stress or alkaline salt stress, of which alkaline salt stress is mainly caused by NaHCO_3_ and Na_2_CO_3_. Alkaline damage, including osmotic, ionic and high pH damage, produces more direct toxic effects on plants than neutral salt stress [3, 4]. Osmotic stress and ionic stress lead to the accumulation of reactive oxygen species (ROS) and trigger enzymatic and nonenzymatic systems to mitigate ROS stress [5, 6]. Excess soluble ions reduce the water potential on the root surface, produce osmotic stress and lead to water shortage in plants [7, 8]. Additionally the elevated pH suppresses photosynthesis, N metabolism, glycolysis and growth of maize plants more markedly than NaCl stress [9]. Seedling emergence and establishment of *Phaseolus vulgaris* were also markedly suppressed under NaHCO_3_ stress [10].

When plants are exposed to alkaline salt stress, the root is the first tissue affected, and various biochemical and physiological mechanisms are stimulated to deal with the stress [11]. Alkaline stress greatly reduces root growth and root vigour, and induces a marked accumulation of superoxide anions (O_2_^.-^) and H_2_O_2_ in rice roots [12]. The increasing pH around the root system also leads to the deposition of metal ions, resulting in the reduction of inorganic anions and hindrance of plant uptake of mineral nutrients [13]. The roots minimize the distribution of absorbed salt at the tissue and cellular levels to avoid accumulation of toxic concentrations in the cytosol of functional leaves [14]. Wheat roots exhibited greater growth performance in response to alkaline salt stress as a result of increased glutamine synthetase activity and soluble protein contents [15].

Plants respond to alkaline salt stress by regulating the expression of a variety of salt-responsive genes, such as osmoregulatory genes [16], transporters/antiporters [17], transcription factors (TFs) [18] and protein kinases (PKs) [19, 20]. Genes encoding Na^+^ transport proteins are involved in regulating Na^+^ transport under alkaline salt stress [21]. A quantitative trait locus (QTL) detected from NaCl and NaHCO_3−_treated rice suggested that the genes controlling the transport of Na^+^, in the form of NaCl and NaHCO_3_, may be different or induced in an uncoordinated manner by salt stress [22]. The vacuolar proton pump ATPase (V-H^+^-ATPase) is a multisubunit membrane protein complex that plays a major role in the activation of ion and nutrient transport. Overexpression of *ScVHA-B*, *ScVHA-C* and *ScVHA-H* improves tolerance to alkaline salt stress in transgenic alfalfa [23]. The HD-Zip TF family is one of the largest plant-specific TF superfamilies and plays important roles in the response to abiotic stresses. *Gshdz4*, an HD-Zip gene, showed a strong response to alkaline stress in wild soybean treated with 50 mM NaHCO_3_ [24]. *MsCBL4* plays an important role in alkaline salt stress tolerance via its influence on the regulation of calcium transport and accumulation [25]. Overexpression of *GsMSRB5a* and *GsCBRLK* in Arabidopsis enhanced alkaline stress tolerance, inhibiting ROS accumulation and modifying the expression of ROS signalling, biosynthesis and scavenging genes. With the continuous development of high-throughput sequencing, some genes and TFs have been identified in switchgrass [26, 27]. These interacting genes form multiple pathways, such as the salt overly sensitive (SOS) pathway, the calcium-dependent protein kinase (CDPK) pathway and the mitogen-activated protein kinase (MAPK) pathway [28].

Switchgrass (*Panicum virgatum* L.), a perennial warm-season C_4_ rhizomatous bunchgrass, shows great prospects in terms of its strong adaptability, high water and nitrogen use efficiency, rapid growth and high productivity [29]. As an important ethanol bioenergy plant, switchgrass is considered an ideal plant to alleviate soil salinization and can still grow well in alkaline saline and marginal soil [30]. Anderson et al. showed that three lowland type switchgrass varieties (EG 2101, EG 1101 and EG 1102) had higher emergence rates and biomass yields under moderate to severe salt stress [29]. However, the molecular mechanism of switchgrass tolerance to alkaline salt stress is not well understood.

There may be much potential to exploit the responses and molecular mechanisms in switchgrass under alkaline salt stress, but the transcriptional regulation and functional genes playing a key role in alkaline salt stress tolerance have remained undescribed. Here the gene expression dynamic and act network were revealed by assaying the physiological and transcriptome changes in roots of two switchgrass genotypes, Alamo (alkaline-tolerant genotype) and AM-314/MS-155 (alkaline-sensitive genotype), under alkaline salt stress for 24 h. The pattern of association between differentially expressed genes (DEGs) and physiological changes in response to alkaline salt stress were explored by weighted gene coexpression network analysis (WGCNA). A regulatory network model based on functional genes was established to comprehensively illustrate the alkaline salt stress tolerance mechanism of switchgrass.

## Results

### Changes in the alkaline salt tolerance trait index in two switchgrass genotypes

**Fig. 1:**
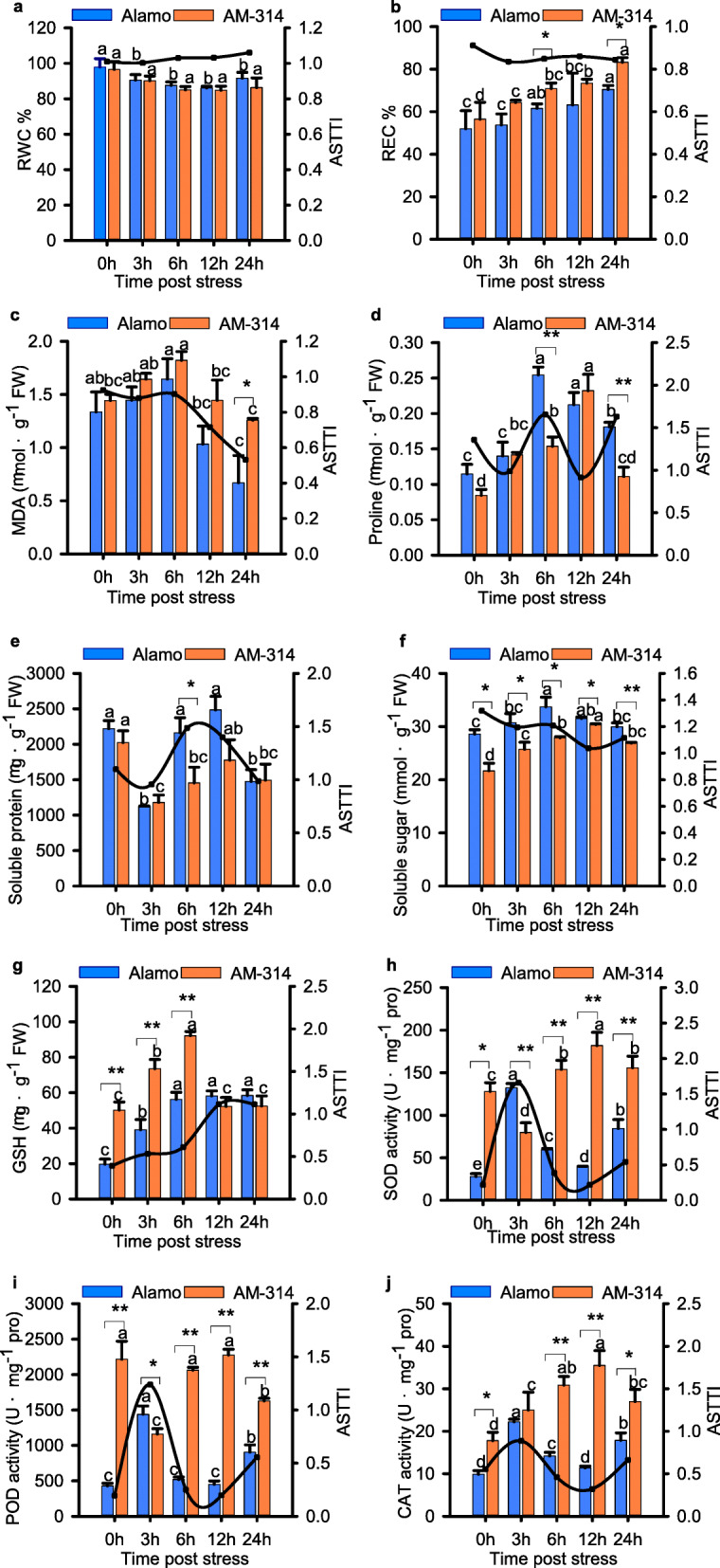
ASTTI evaluation of two lowland switchgrass genotypes (Alamo and AM-314/MS-155) by the physiological changes in roots under alkaline salt stress for 0, 3, 6, 12 and 24 h. **a**: Relative water content (RWC). **b**: Relative electric conductivity (REC).**c**: Malondialdehyde (MDA) content. **d**: Proline content. **e**: Soluble protein content. **f**: Soluble sugar content. **g**: Glutathione content (GSH). **h**: Superoxide dismutase (SOD) activity. **i**: Peroxidase (POD) activity. **j**: Catalase (CAT) activity. The left Y-axis represented value of physiological traitors, and the right Y-axis represented value of ASTTI. The assays were repeated three times with three biological replicates. The data, shown as means ± SEs, were subjected to the Student’s t test and one-way ANOVA with Duncan’s multiple range test to determine significant differences. “*” denotes *p* < 0.05, “**” denotes *p* < 0.01. The different letters represent statistically significant differences within genotype (α = 0.05)

The physiological responses in roots of Alamo (alkaline-tolerant genotype) and AM-314/MS-155 (alkaline-sensitive genotype) under alkaline salt stress for 0, 3, 6, 12 and 24 h were evaluated by the alkaline salt tolerance trait index (ASTTI). Although the relative water content (RWC) decreased under alkaline salt stress, the ASTTI of RWC showed an upward trend (Fig. 1a). The ASTTI values of the relative electrical conductivity (REC) and malondialdehyde (MDA) content showed a downward trend with stress time (Fig. 1b and c). The REC and MDA contents in Alamo were higher than in AM-314/MS-15 at all treatment time points. The significant differences in REC between Alamo and AM-314/MS-15 were observed at 6 and 24 h under alkaline stress. The REC in AM-314/MS-15 were 1.16 and 1.18 times higher than in Alamo, respectively. The root MDA contents in AM-314/MS-155 were 1.88 times higher than in Alamo at 24 h post alkaline salt stress.

The contents of free proline, soluble protein and soluble sugar were used as indicators of the osmotic adjustment system. The ASTTI value of proline and soluble protein contents first increased with increasing stress duration and then decreased, and the value of ASTTI of soluble sugar gradually declined with the increase in stress time (Fig. 1d-f). The proline content in Alamo was significantly higher than in AM-314/MS-155 at 6 and 24 h post alkaline salt stress. Although the contents of soluble protein only showed a significant difference between Alamo and AM-314/MS-15 at 6 h, the contents of soluble sugar showed significantly differences at all treatment times.

The contents of reduced glutathione (GSH) and the activities of superoxide dismutase (SOD), peroxidase (POD) and catalase (CAT) were used as indicators of the antioxidant system (Fig. 1g-j). Interestingly, the ASTTI value of GSH steadily rose under alkaline stress, while the value of ASTTI of SOD, POD and CAT first increased and then decreased and re-increased with stress time. These four physiological traits changed significantly between Alamo and AM-314/MS-15 under alkaline salt stress, especially the activities of SOD and POD, and they changed significantly at all treated time points.

In addition, we observed significant physiological differences between the two varieties at different time points under alkaline stress, especially at 6 and 24 h. Therefore, we chose these two time points for further transcriptome analysis.

### De novo assembly and annotation of unigenes

Six cDNA libraries were constructed from total RNA extracted from E5 stage roots of Alamo and AM-314/MS-155 treated with alkaline salt stress for 0, 6 and 24 h. A total of 114.03 Gb of clean data were obtained, with 6.05 Gb of clean data for each sample and a Q30 base percentage of 93.21% or more (Additional file 1: Table S1). Since the whole-genome sequence of switchgrass is not currently publicly available, valid readings from the six libraries were combined for reassembly (Additional file 2: Table S2). A total of 108,319 unigenes were obtained after reassembly, including 73,636 unigenes in AM-314 and 65,492 unigenes in Alamo. The total length, N50 length and mean length were 106,429,710 nt, 1751 nt and 982.56 nt, respectively. Functional annotation of assembled sequences was primarily based on BLAST homology searches with the NCBI nonredundant protein (Nr), Evolutionary Genealogy of Genes: Nonsupervised Orthologous Groups (eggNOG), Gene Ontology (GO), Pfam, Swiss-Prot, Eukaryotic Orthologue Groups (KOG), Kyoto Encyclopedia of Genes and Genomes (KEGG), and Clusters of Orthologous Groups (COG) databases, and a total of 66,253 genes were annotated (Additional file 3: Table S3). Among these annotated sequences, 63,663 (96.09%) sequences had significant matches in the Nr database. On the basis of the homology among sequences of different species, 25,805 (40.56%) sequences were found against *Setaria italica,* and 5124 (8.05%) sequences had clandestine hits with *Sorghum bicolor*, followed by *Zea mays* (4732, 7.44%), *Oryza sativa* (1590, 2.50%), *Phaeosphaeria nodorum* (1564, 2.46%), *Verticillium dahliae* (950, 1.49%), *V. alfalfae* (839, 1.32%), *Phytophthora sojae* (680, 1.07%), *Togninia minima* (627, 0.99%) and *Hordeum vulgare* (584, 0.92%). Only 21,129 (33.21%) annotated sequences were similar to those of other plant species (Additional file 4: Fig. S1A). The gene expression levels in response to alkaline salt stress were evaluated by converting the mapped read count for each gene into the expected number of fragments per kilobase of reproduction per million mapped reads (FPKM) to eliminate the effects of transcript length and sequencing differences on computational expression (Additional file 5: Table S4). The gene expression levels were not evenly distributed in the different stress environments in the boxplot diagram of the FPKM values (Additional file 4: Fig. S1B). Then, the correlations of each biological sample were evaluated by Pearson correlation coefficients, and a value of r^2^ close to 1 indicated a strong correlation between two replicate samples (Additional file 4: Fig. S1C). Finally, all the unigenes were used for further identification of DEGs after the exclusion of abnormal samples.

### Identification of DEGs associated with alkaline salt stress

To identify the DEGs of the two different genotypes under alkaline stress, the expression patterns of DEGs were analysed by comparing 6-h and 24-h libraries with the control library for Alamo and AM-314/MS-155. A total of 10,219 DEGs with up- or downregulated expression between samples (fold change ≥2 and false discovery rate (FDR) < 0.01) at any pair of alkaline salt-treated points were identified (Additional file 6: Table S5). In the early stage of alkaline salt stress (0–6 h), 4483 genes (58.02% of the DEGs) were upregulated in AM-314/MS-155, and 867 genes (61.36% of the DEGs) were upregulated in Alamo (Table 1). In the late stage of alkaline salt stress (24 h), 2942 genes (56.17% of the DEGs) were upregulated in AM-314/MS-155, while Alamo had 2732 upregulated genes (61.61% of the DEGs). The proportion of upregulated genes in the tolerant genotype (Alamo) was higher than that in the sensitive genotype (AM-314/MS-155) in both the early and late stages of stress. In addition, the DEGs of AM-314/MS-155 were mainly concentrated in the early stage, while Alamo showed greater advantages in the late stage.

**Table 1:** Comparison of DEGs between two genotypes at different time points

Items	AM-314/MS-155 (sensitive)			Alamo (tolerant)		
	0 h vs 6 h	0 h vs 24 h	6 h vs 24 h	0 h vs 6 h	0 h vs 24 h	6 h vs 24 h
Total	77,578	83,255	34,658	78,349	77,799	33,172
DEGs	7727	5238	1150	1413	4434	1255
Up-regulated	4483	2942	361	867	2732	675
Down-regulated	3244	2296	789	546	1702	580

**Fig. 2:**
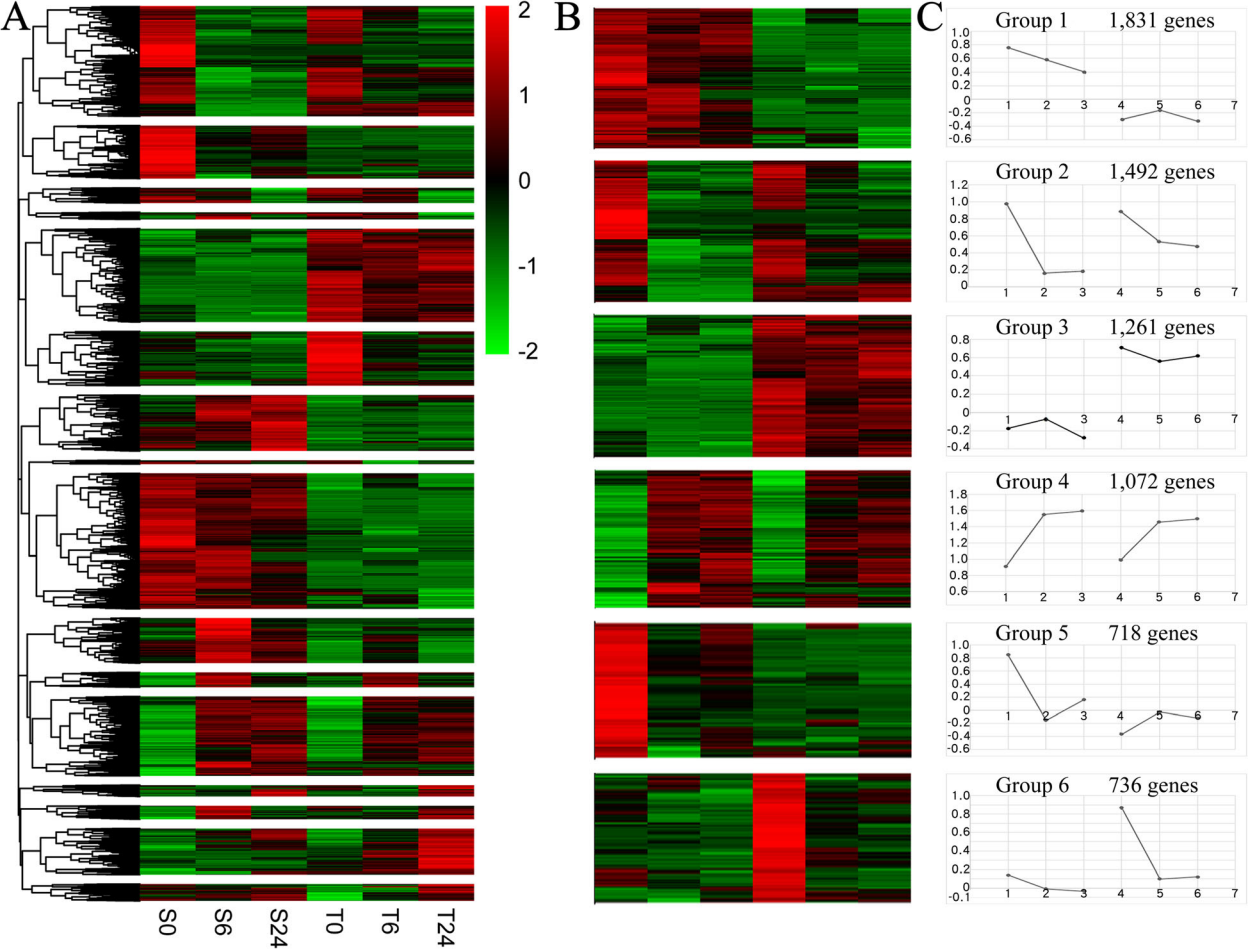
Clustering model analysis of DEGs. **a**: The 16 clustering modules, in which S0, S6 and S24 indicate the DEGs of alkaline-sensitive genotype AM-314/MS-155 under alkaline salt stress for 0, 6 and 24 h, while T0, T6 and T24 denote the DEGs of alkaline-tolerant genotype Alamo under alkaline-salt stress for 0, 6 and 24 h. **b**: Clustering of the mainstream genes. **c**: The changing trend of the mainstream genes of two genotypes with stress time

To further analyse the effects of alkaline salt treatment in the two genotypes over time, the expression trends of DEGs were clustered into 16 modules (Fig. 2a). Then, the mainstream gene expression trends (6 groups) were screened over the duration of the stress (Fig. 2b). The first group contained 1831 enriched genes, which mainly reflected the functional classification of genes expressed abundantly in AM-314/MS-155 but with little or no expression in Alamo (Fig. 2c). A large number of ribosomal proteins and a small number of cytochrome and energy-related genes were enriched in the ribosome and oxidative phosphorylation pathways and gradually downregulated with increasing stress time (Additional file 7: Table S6). The expression level of genes in group 2 (1492 genes) decreased sharply in the early stage of alkaline salt stress in both switchgrass genotypes. KEGG analysis of the genes in group 2 revealed that most were involved in the ribosome, phenylalanine metabolism, plant hormone signal transduction and ribosome biogenesis in eukaryote pathways (Additional file 8: Table S7). The expression of genes in group 3 (1261 genes) was completely opposite to that in group 1, mainly reflecting the functional classification of genes expressed abundantly in Alamo but with little or no expression in AM-314/MS-155. Genes in this group functioned mostly in the glycolysis/gluconeogenesis, plant-pathogen interaction, amino sugar and nucleotide sugar metabolism and ubiquitin-mediated proteolysis categories (Additional file 9: Table S8). The expression of genes in group 4 (1072 genes) increased strongly in the early stage of alkaline salt stress, and most of these genes functioned in starch and sucrose metabolism, valine, leucine and isoleucine degradation, plant hormone signal transduction, galactose metabolism and fatty acid degradation (Additional file 10: Table S9). Despite a rapid decrease in gene expression levels in both group 5 (718 genes) and group 6 (736 genes), the decrease in the expression of genes in group 5 was mainly in AM-314/MS-155, while the decrease in the expression of genes in group 5 was mainly in Alamo. Genes involved in oxidative phosphorylation, protein processing in the endoplasmic reticulum, glycolysis/gluconeogenesis and glycerophospholipid metabolism were enriched in group 5 (Additional file 11: Table S10), and genes in group 6 were enriched in ribosome, oxidative phosphorylation, glycolysis/gluconeogenesis and DNA replication (Additional file 12: Table S11).

### Identification of TFs and PKs

In this study, 1480 TF genes were predicted using ITAK software (http://itak.feilab.net/cgi-bin/itak/index.cgi) and were classified into 64 TF families (Additional file 13: Table S12). The most abundant TF families were the C2H2 (153 genes), bZIP (108 genes), bHLH (93 genes), C3H (91 genes), MYB-related (82 genes), NAC (81 genes), WRKY (74 genes), GRAS (57 genes) and AP2/ERF-ERF (52 genes) families. The genes in the bHLH, C2H2, NAC and WRKY families were mainly upregulated under alkaline stress, while the genes in the AP2/ERF-ERF, bZIP and MYB-related families showed a relatively balanced number of upregulated and downregulated members (Additional file 14: Fig. S2). There were 32 TF genes that showed differential expression in both Alamo and AM-314/MS-155 under alkaline salt stress, including 7 TF genes that showed differential expression only at 6 h, 10 TF genes that showed differential expression only at 24 h and 15 TF genes that showed differential expression at both 6 and 24 h (Additional file 15: Table S13). Interestingly, the TF genes that were only differentially expressed at 6 h were all downregulated, and most of them belonged to the MYB-related family. Nine of ten TF genes that were differentially expressed only at 24 h were unregulated, and most of them belonged to the NAC family. There were 27 upregulated TF genes and 16 downregulated TF genes specifically expressed in Alamo, and many of them were differentially expressed in the late stage of alkaline stress (24 h) and belonged to the bZIP, MYB-related and bHLH families (Additional file 16: Table S14).

In addition, a total of 1718 PKs, such as calcium/calmodulin-dependent protein kinase (CaMK), CDPK, MAPK and receptor-like kinase (RLK), were predicted in AM-314/MS-155 and Alamo under alkaline salt stress for 6 and 24 h (Additional file 17: Table S15). The CBL-interacting protein kinases (CIPKs), which play a role as Ca^2+^ sensors, were upregulated and highly expressed in Alamo under alkaline salt stress for 24 h. RLKs accounted for the largest proportion among many protein kinase families, and the genes belonging to the RLK family maintained a high expression level at 6 h and were then downregulated at 24 h.

### Weighted Gene Coexpression Network Analysis (WGCNA) of DEGs in response to alkaline salt stress

**Fig. 3:**
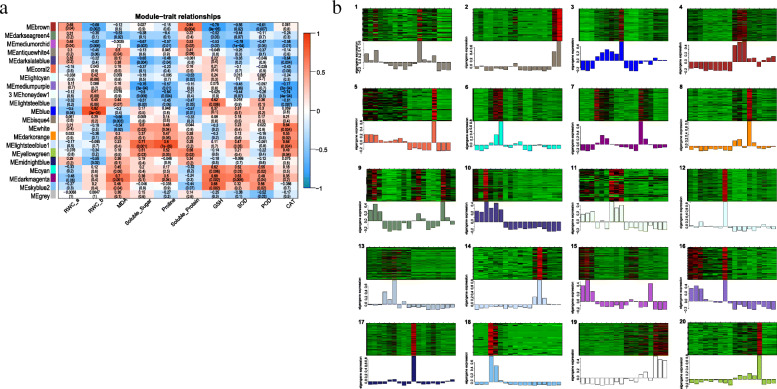
Correlation analysis between modules and physiological traits by WGCNA (**b**), and eigengenes expression patterns of 20 modules of WGCNA clustering (**b**). Each row corresponds to a clustering module, each column corresponds to a physiological trait, the colour of each grid at the row-column intersection indicates the correlation between the module and physiological data, red represents a positive correlation, blue represents a negative correlation, and the number in each grid represents the correlation coefficient and the *P* value. Twenty modules were marked with numeric value. 1: antiquewhite4; 2: bisque4; 3: blue; 4: brown; 5: coral2; 6: cyan; 7: darkmagenta; 8: darkorange; 9: darkseagreen4; 10: darkslateblue; 11: honeydew; 12: lightcyan; 13: lightsteelblue; 14: lightsteelblue1; 15: mediummorchid; 16: mediumpurple3; 17: midnightblue; 18: skyblue2; 19: white; 20: yellowgreen. In each module, red represents up-regulation and green represents down-regulation. The following corresponds to the degree of up-and-down adjustment of 18 samples in 6 groups (S0, S6, S24, T0, T6, T24)

To further study the patterns of association between the DEGs responding to alkaline salt stress and physiological indicators across the two genotypes, WGCNA was performed to explore the gene modules for synergistic expression. After the filtering of low-expression genes (FPKM < 5), the remaining 7485 genes were classified into 20 different modules (combined modules) (Fig. 3a).

**Fig. 4:**
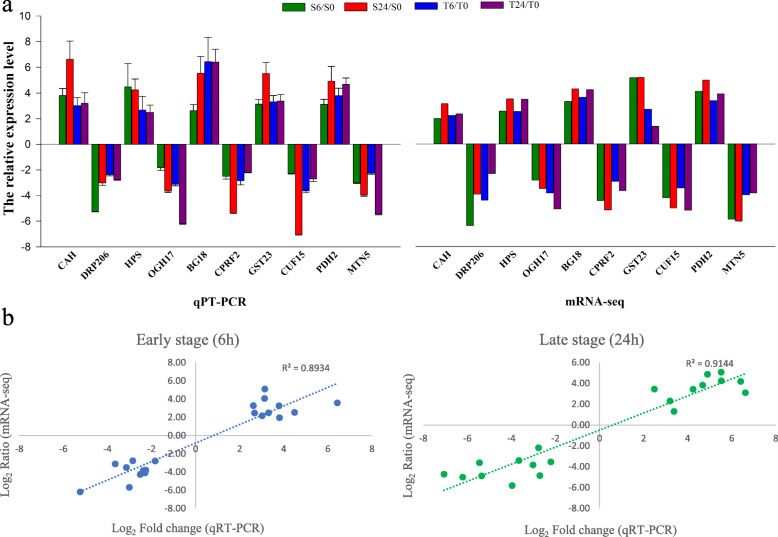
Verification of RNA-Seq sequencing data by the qRT-PCR assay (**a**) and Pearson correlation coefficients of genes under alkaline-salt stress for 6 and 24 h (**b**). The Y-axis represents qRT-PCR relative expression levels from three biological replicates and the log_2_ fold-change of the unigenes

The blue module showed the highest positive correlation with the REC (R = 0.82, *p* = 3e-5) (Fig. 3b). This module was characterized by a significant upregulation of DEGs in the alkaline-sensitive genotype AM-314/MS-155 at 6 and 24 h (Fig. 3c). Genes in this module were significantly enriched in the peroxisome, amino sugar and nucleotide sugar metabolism, starch and sucrose metabolism and plant-pathogen interaction pathways by KEGG (Additional file 18: Fig. S3A). The brown module showed a positive correlation with soluble protein (R = 0.64) and the RWC (R = 0.49). This module represented the downregulated genes in Alamo under alkaline salt stress. The functions of these genes were mainly concentrated in the plant hormone signal transduction, phenylpropanoid biosynthesis and biosynthesis of amino acid pathways (Additional file 18: Fig. S3B). The dark magenta module showed a high positive correlation with MDA, GSH and SOD, with R values of 0.70, 0.68 and 0.63, respectively. This module showed the features of significantly upregulated genes in both Alamo and AM-314/MS-155 under alkaline salt stress for 6 h. Genes in this module were mainly enriched in the amino sugar and nucleotide sugar metabolism, starch and sucrose metabolism, and other functional pathways (Additional file 18: Fig. S3C). The light steel blue 1 module was positively correlated with proline, soluble sugar and CAT, with R values of 0.80, 0.71 and 0.64, respectively (Fig. 4b). The module mainly showed genes in the alkaline-tolerant Alamo that were significantly upregulated under alkaline salt stress for 6 h. These genes were mainly enriched in the functions of phenylalanine metabolism and phenylpropanoid biosynthesis (Additional file 18: Fig. S3D).

### Verification of RNA-Seq sequencing data by qRT-PCR analysis

The DEGs associated with alkaline salt stress were selected for qRT-PCR assays to verify the accuracy of the RNA-Seq sequencing data. Ten genes were selected randomly from the DEGs that were coexpressed in all sequencing samples. The expression patterns of these ten genes were the same as those of the RNA-Seq assay (Fig. 4a). The expression levels indicated by the qRT-PCR results were strongly correlated with the differential gene expression levels identified by mRNA-seq according to the Pearson correlation coefficients, which were 0.8934 and 0.9144 under alkaline salt stress for 6 and 24 h, respectively (Fig. 4b), and demonstrated the reliability of the RNA-seq data.

## Discussion

### Comparison of physiological differences between different switchgrass genotypes

Due to the combined effects of osmotic, ionic and secondary stresses, plant growth is severely affected by alkaline salt stress [7]. With the prolongation of alkaline stress, the cell membrane is destroyed, resulting in increased membrane permeability and severe lipid peroxidation, and alkaline-tolerant genotypes show an obvious advantage [31]. In our study, the root REC and MDA content in AM-314/MS-155 were significantly higher than those in Alamo at 24 h post alkaline salt stress, which indicated that Alamo was more alkaline salt tolerant than AM-314/MS-155, and the degree of damage in Alamo was gradually reduced compared with AM-314/MS-155.

The accumulation of osmotic adjustment substances, such as proline, soluble protein and soluble sugar, is an important factor in sustaining plant growth under alkaline salt conditions [32]. The relative alkaline-tolerant varieties resist alkaline salt stress by enhancing compatible solutes, including proline, thus maintaining cell turgor and water potential to achieve better growth and development [33]. In our study, the accumulation of soluble proteins was relatively lagging, which indicated that proline and soluble sugar could be used as important osmotic regulators in switchgrass under alkaline treatment.

Antioxidant enzymes are the most important components of the ROS scavenging system. SOD can catalyse the formation of hydrogen peroxide and oxygen by superoxide radicals, and POD and CAT further scavenge H_2_O_2_ [34]. In this paper, the SOD activity of the alkaline-tolerant Alamo increased rapidly at 3 h. The expression patterns of POD and SOD induced by alkaline stress were similar in switchgrass. The nonenzymatic substance GSH in the antioxidant system also has the effect of scavenging ROS, and the glutathione transferase (GST) family is also a very important component of the metabolic detoxification enzyme system [35]. The GSH content of the sensitive genotype AM-314/MS-155 was significantly higher than the alkaline-tolerant genotype Alamo in the early stage of alkaline salt stress. We found that the glutathione S-transferase genes in sensitive cultivars were significantly upregulated in the early stage of alkaline salt stress, and this may have been controlled by the regulation of the related genes, leading to changes in physiological indicators. These results revealed the physiological responses of switchgrass to complex alkaline salt stress, including the initial injury accumulation phase (0–6 h) and subsequent gradual recovery of infiltration and ion homeostasis phases (6–24 h).

### Gene expression in response to alkaline salt stress in two switchgrass genotypes

In this study, a total of 10,219 DEGs were identified in two switchgrass genotypes under alkaline salt stress. The proportion of upregulated genes in Alamo was higher than that in AM-314/MS-155, and the DEGs in AM-314/MS-155 were mainly concentrated in the early stage, suggesting quick adjustments in dealing with the damage caused by alkaline salt stress in the short term. All DEGs clustered into 16 modules, and the mainstream gene trends were grouped into six groups (Fig. 2). Thirteen genes were enriched in the glycolysis/gluconeogenesis pathway, and five of them were hardly expressed in Alamo. Glycolysis/gluconeogenesis was considered to be the key pathway in the process of root development because the component exchanges with other pathways [36]. These results suggested that the sensitive genotype was not resistant to alkaline stress in the late stage. Some disease resistance proteins were enriched in the plant-pathogen interaction pathway; they all contained the NB-ARC domain, which plays an important role in plant hypersensitivity, a prerequisite for the initiation of defence responses [37, 38].

### Identification of alkaline-tolerant TFs in two switchgrass genotypes

In this study, bHLH, bZIP, C2H2 and WRKY were significantly enriched at 6 h in AM-314/MS-155, while bZIP, MYB-related and NAC were significantly enriched at 24 h in Alamo. The specific upregulated TFs associated with high alkaline tolerance in Alamo were AP2-ERF, bZIP, bHLH and MYB-related. AP2-ERF transcription factors are involved in many physiological processes, including plant growth [39], abiotic stress responses [40] and ethylene and abscisic acid signalling pathways [41]. It was reported that *Gm-ERF3* isolated from soybean could improve the salt tolerance of transgenic tomato [42]. Overexpression of *PvERF001* isolated from the ERF genes of switchgrass could also increase biomass yield and sugar release efficiency in transgenic lines [43]. In this paper, we observed that AP2-ERF family TFs were still significantly upregulated at 24 h and might be used as candidate TFs to improve alkaline tolerance in switchgrass. Overexpression of bZIP and bHLH TFs can help plants positively respond to salt stress [44]. Rong et al showed that *OsMYBc* in rice can regulate *OsHKT1* to deal with Na^+^ damage caused by salt stress and prevent leaf accumulation of Na^+^ toxicity [45]. In our study, the bZIP and MYB TFs continuously positively responded to alkaline salt stress at 6 h and 24 h. The bHLH TFs were specifically upregulated at 6 and 24 h in Alamo, indicating that the alkaline salt tolerance of Alamo and these TFs could be considered target genes involved in the alkaline stress response.

### Identification of functional genes and potential regulatory response mechanisms of alkaline tolerance in switchgrass under alkaline salt stress

Under alkaline salt stress, the genes encoding transporters responsible for reconstituting osmotic and ion-balanced proteins under alkaline stress, such as sodium/hydrogen exchange protein (NHX), alkaline and neutral invertase (CINV2) and heat shock proteins (HSPs) including HSP20, HSP70, Hsp90 and DanJ, were differentially expressed in the two switchgrass genotypes. The ABC transporter and H^+^ transport ATPase genes were significantly upregulated at 6 and 24 h in the two genotypes and showed a downward trend in Alamo in the late stage. Simultaneously, osmotic and ionic stresses can also lead to an increase in the concentration of free Ca^2+^ in the cytosol, which results in the downstream gene phosphorylation and protein cascade [46]. The CDPK and MAPK pathways are important signalling pathways in plant responses to salt stress [47]. The MAPK cascade is an important regulator of antioxidant defence [48]. CAMS/CMLS, CIPKs and CDPKs, which function as Ca^2+^ sensors, are involved in many growth, development and stress-induced signal transduction pathways [49, 50]. Overexpression of *OsCIPK1* and *OsCIPK9* not only occurs in response to salt stress, but also to ABA, drought and cold stress [51]. In this study, MAPK6 was significantly upregulated at 6 and 24 h in the two genotypes. CIPKs were mainly upregulated at 6 and 24 h in Alamo, while expression in AM-314/MS-155 remained low. These results suggest that the CDPK and MAPK signalling pathways may also mediate the response of switchgrass to alkaline stress.

Compared with the two genotypes, many more DEGs were involved in plant hormone signal transduction in AM-314/MS-155 than in Alamo at 6 h, while more DEGs were identified in Alamo at 24 h. Alamo showed differential expression in the ABA signalling pathway at two different time points, while AM-314/MS-155 showed no significantly enriched DEGs. ABA plays a very important role in plant salt-tolerance signal transduction pathways and participates in the physiological and biochemical processes of abiotic stress in plants [52]. In our study, the HVA22 protein, a unique ABA stress-induced protein, was mainly upregulated in Alamo at 24 h and in AM-414/MS-155 at 6 h under alkaline salt stress. The HVA22 protein is induced by environmental stresses such as drought and high salt, which could inhibit GA-mediated programmed cell death of cereal aleurone cells and improve plant resistance to stress [53].

ROS also act as second messengers [54, 55]. Detoxification proteins involved in ROS scavenging were highly expressed at 6 and 24 h. Some DEGs were expressed more in AM/314/MS-155 than in Alamo, but most of the favourable upregulated genes were specifically expressed in the alkaline-tolerant genotype Alamo. LEA proteins have high hydrophilicity and can supplement cells with sufficient water during stress and lessen the damage caused by osmotic stress [56]. Dehydrin, a second type of LEA protein, can significantly increase the resistance of plants to high salt and osmotic stress [57]. In this paper, the LEA protein was downregulated in AM-314/MS-155 but upregulated in Alamo. The dehydrin protein showed specific upregulation only in Alamo, which fully reflected the positive response of Alamo to alkaline stress.

Alkaline/neutral sucrase genes were also upregulated at 6 and 24 h under alkaline salt stress, and the number and expression of genes upregulated were higher in Alamo than in AM-314/MS-155. Alkaline/neutral sucrase is a class of genes unique to plants and photosynthetic bacteria. The total alkaline/neutral invertase activity in wheat leaves increases after osmotic or low-temperature stress, resulting in increased expression of related genes [58].

**Fig. 5:**
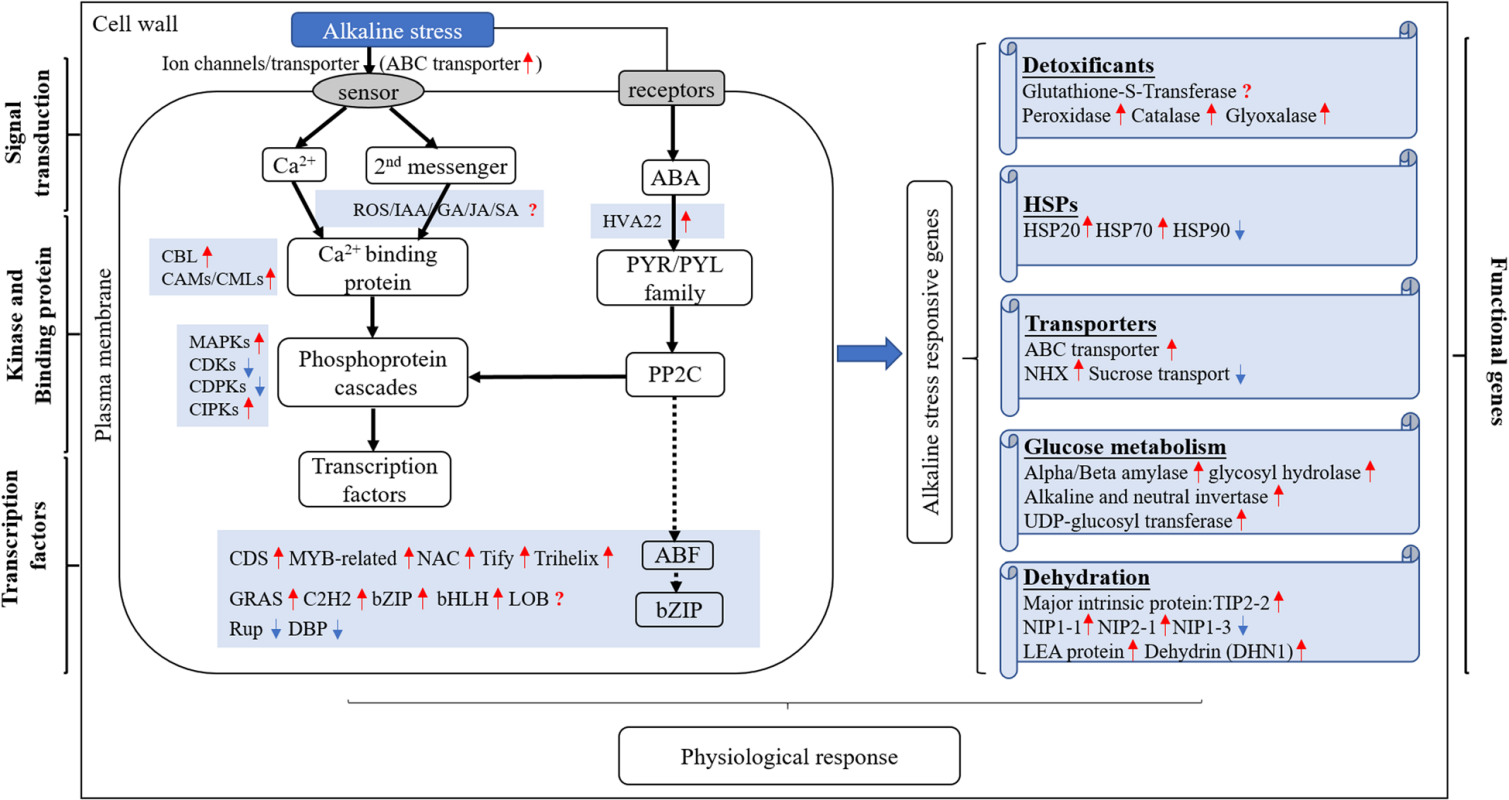
Possible molecular mechanisms of switchgrass in response to alkaline salt stress. The red-up arrows indicate genes that were up-regulated, the blue-down arrows indicate genes that were down-regulated and the “?” denotes the expression of conflicting genes, or both up- or down-regulated under alkaline salt stress in roots of two switchgrass genotypes

These genes are involved in antioxidant systems and transport functions and can aid in plant adaptation to alkaline saline environments by regulating ion homeostasis, transport proteins, detoxification, heat shock proteins, dehydration and sugar metabolism. The interaction of these genes constituted a complex regulatory network (Fig. 5). We hypothesized that switchgrass plants respond to alkaline salt stress through ion and osmotic stress signals. These signals were first sensed by receptors present on the plant cell membrane and then involved in signal transduction through ion channels and carrier proteins. The receptors in the roots may recognize salt signals, causing an instantaneous increase in ROS, leading to the opening of some plant hormone signalling pathways (including those of auxins, abscisic acid, cytokinins, gibberellins, ethylene, jasmonic acid and salicylic acid), biosynthesis of ubiquinone and other terpenoid-quinones, phenylpropanoid metabolism and biosynthetic pathways.

## Conclusions

Taken together, we investigated the alkaline salt stress tolerance mechanism of switchgrass by analysing gene expression dynamic and act network in two switchgrass genotypes (AM-314/MS-155 and Alamo). A total of 108,319 unigenes were obtained after reassembly, and 10,219 DEGs were identified in the two switchgrass genotypes under alkaline salt stress. The DEGs in AM-314/MS-155 were mainly concentrated in the early stage, while the DEGs in Alamo showed greater advantages in the late stage. We characterized 1480 TF genes and 1718 PKs, and the most abundant TF family was the C2H2 family. The WGCNA results revealed that the DEGs in the blue, brown, dark magenta and light steel blue 1 modules were associated with the physiological changes in roots of switchgrass under alkaline salt stress. The alkaline salt tolerance of switchgrass may be achieved by the regulation of ion homeostasis, transport proteins, detoxification, heat shock proteins, dehydration and sugar metabolism.

## Methods

### Plant materials and growth conditions

Seeds of two lowland switchgrass (*Panicum virgatum* L.) genotypes (the alkaline salt tolerant genotype ‘Alamo’ and sensitive genotype ‘AM-314/MS-155’) were used to conduct this research. These two switchgrass lines were originally obtained from the United States Department of Agriculture Germplasm Center, and the accession number was ‘PI 422006’ for Alamo and ‘PI 421999’ for AM-314/MS-155. The alkaline salt tolerance of these two lines were evaluated by Hu et al. [59]. Hand-selected seeds of Alamo and AM-314/MS-155 were surface-sterilized in 5% sodium hypochlorite for 3 h and rinsed 4 to 5 times with sterile distilled water. Then, the seeds were germinated in culture dishes with wet filter papers in a dark growth chamber at 25 °C. Five-day-old seedlings were transported to plastic pots (17.8 cm upper diameter, 10 cm bottom diameter and 23.8 cm deep, with 8 drainage holes at the bottom) with the bottom 1/4 filled with coarse sand and the top 3/4 of the pots filled with fine sand in a greenhouse. Seedlings were kept in the greenhouse for 12 weeks (daytime temperature was 35 ± 1 °C, night-time temperature was 25 ± 1 °C, 16 h of light and relative humidity of 70–80% RH) and fertilized with half-strength Hoagland’s nutrient solution every 2 days.

### Alkaline salt stress treatments

Alkaline salt stress treatments were conducted when the seedlings reached the E5 developmental stage [60]. The alkaline salt solution, which was made of Na_2_CO_3_ and NaHCO_3_ (1:9, v/v), with Na^+^ at 150 mM and pH 9.5, was prepared with half-strength Hoagland’s nutrient solution [59]. Roots of the two genotypes were harvested at 0, 3, 6, 12 and 24 h post alkaline salt stress. The experiment was repeated three times with three biological replicates. For each biological replicate, some of the samples were used to determine the relative water content (RWC) and relative electric conductivity (REC) directly, and the rest were frozen in liquid nitrogen immediately and stored at − 80 °C for physiological and transcriptome analyses.

### ASTTI evaluation by physiological assays

Physiological traits including the RWC, REC, the contents of MDA, free proline, soluble protein, soluble sugar and reduced GSH, and the activities of SOD, POD and CAT in roots of two switchgrass genotypes at the E5 developmental stage were assayed three times with three biological replicates [61–64]. The ASTTI, which was calculated based on the formula, ASTTI = (value of a trait under salt stress condition)/(value of a trait under controlled condition), was used to estimate the alkaline salt tolerance of the two switchgrass genotypes [59, 65].

### RNA extraction, library preparation and Next-Generation Sequencing (NGS) analysis

The roots of Alamo and AM-314/MS-155 at the E5 developmental stage under alkaline salt stress for 6 and 24 h were chosen for transcriptome analysis. Total RNA was extracted with TRIzol reagent (Invitrogen, USA) according to the manufacturer’s protocol, and genomic DNA was removed via digestion with DNase I (TaKaRa, Japan). The experiment was repeated three times with three biological replicates. The RNA sample concentration and quality were determined using a Nanophotometer P330 spectrophotometer (IMPLEN, Germany). A total amount of 3 μg RNA per sample was used as input material for the RNA sample preparations. Sequencing libraries were generated using the NEBNext®Ultra™ RNA Library Prep Kit for Illumina® (NEB, USA) following the manufacturer’s recommendations, and index codes were added to attribute sequences to each sample.

The clustering of the index-coded samples was performed on a cBot Cluster Generation System using the TruSeq PE Cluster Kit v3-cBot-HS (Illumina) according to the manufacturer’s instructions. After cluster generation, the library preparations were sequenced on an Illumina HiSeq 2000 platform, and paired-end reads were generated. Clean reads were obtained by removing reads containing adapters, reads containing poly-N and low-quality reads from the raw data. All downstream analyses were based on clean data with high quality. Transcriptome assembly was accomplished using Trinity Software with min_kmer_cov set to 2 by default and all other parameters set to the default selections [66]. The clean reads of each sample were sequence aligned with the assembled unigene library, and the transcriptome sequencing library was evaluated for quality. Gene function was annotated using BLAST software based on the NR, Pfam, KOG, COG, eggNOG, Swiss-Prot, KEGG and GO databases [67].

### Identification of DEGs, putative TFs and PKs

The paired reads were compared with the unigene library using Bowtie [68] and RNA-seq by expectation maximization (RSEM) [69], and the expression abundance of a unigene was expressed using the FPKM value [70]. Differential expression analysis under the two conditions was performed using the DESeq R package (1.10.1). A false discovery rate (FDR) < 0.01 and fold change (FC) ≥2 were used as screening criteria, and samples with a strong correlation between samples were selected for subsequent functional expression annotation and enrichment analysis of the DEGs. GO enrichment analysis of the DEGs was implemented by the topGO R package-based Kolmogorov–Smirnov test. KOBAS software was subsequently used to test the statistical enrichment of DEGs in the KEGG pathways [71]. TFs and PKs were predicted using ITAK software (http://itak.feilab.net/cgi-bin/itak/index.cgi).

### Coexpression network analysis with WGCNA

Coexpression networks were constructed via the WGCNA package in R from all the DEGs [72]. Modules were obtained by the automatic network construction function using blockwise modules with default settings. The eigengene value was calculated for each module and used to test the association with each physiological index. The total connectivity and intramodular connectivity (function soft connectivity), kME (for modular membership) and kME *p* values were calculated for the DEGs [73].

### Validation of NGS data by quantitative real-time PCR

Total RNA was extracted from roots with TRIzol (Invitrogen, USA) from the same group of samples that were used in the transcriptome analysis, and cDNA synthesis was performed with SuperMix for qPCR (+gDNA wiper) (Vazyme, China) according to the manufacturer’s instructions. Primers used in this study (Additional file 19: Table S16) were designed using primer-BLAST (https://www.ncbi.nlm.nih.gov/tools/primerblast/index.cgi?LINK_LOC=BlastHome). The expression of *CYP-5* was used as an internal control [74]. qPCR was performed using ChamQ™ Universal SYBR qPCR Master Mix (Vazyme, China) on a LightCycler 480 II58 device (Roche, Switzerland) according to the manufacturers’ protocol. The experiment was repeated three times with three biological replicates. Relative gene expression levels were evaluated according to the 2^-ΔΔCT^ method [75]. Each gene was tested in triplicate with three biological replicates.

## Supplementary Information

**Additional file 1: Table S1.** Statistics of sample sequencing evaluation data.

**Additional file 2: Table S2.** Statistics of reassembly results.

**Additional file 3: Table S3.** Statistics of unigene annotations.

**Additional file 4: Figure S1.** Nr homologous species distribution.

**Additional file 5: Table S4.** FPKM values of all unigenes.

**Additional file 6: Table S5.** Information on the 10,219 DEGs under alkaline salt stress.

**Additional file 7: Table S6**. Information on the 1831 genes in Group 1.

**Additional file 8: Table S7**. Information on the 1492 genes in Group 2.

**Additional file 9: Table S8**. Information on the 1261 genes in Group 3.

**Additional file 10: Table S9**. Information on the 1072 genes in Group 4.

**Additional file 11: Table S10**. Information on the 718 genes in Group 5.

**Additional file 12: Table S11**. Information on the 736 genes in Group 6.

**Additional file 13: Table S12**. Summary of the 1480 TFs in response to alkaline salt stress.

**Additional file 14: Figure S2.** Families of differentially expressed TFs under alkaline salt stress. The X-axis represents families of TFs that were differentially expressed in the four comparisons, and the Y-axis represents the number of differentially expressed (up- or down-regulated) genes in each family. The four comparisons were S6/S0, S24/S0, T6/T0 and T24/T0, where S and T represents AM-314/MS-155 and Alamo, treated with alkaline stress treatment for either 6 or 24 h compared with control (0 h), respectively.

**Additional file 15: Table S13.** Summary of differentially expressed TF genes in both AM-314/MS-155 (S) and Alamo (T) under alkaline salt stress.

**Additional file 16: Table S14.** Summary of differentially expressed TF genes only in Alamo (T) under alkaline salt stress.

**Additional file 17: Table S15.** Summary of 1718 protein kinases in response to alkaline salt stress.

**Additional file 18: Figure S3.** Functional enrichment analysis of modules with a higher physiological correlation. A: blue module, B: brown module, C: darkmagenta module, and D: lightsteelblue 1 module.

**Additional file 19: Table S16.** List of primers used in qRT-PCR.

## Data Availability

All relevant supplementary data are provided within this manuscript as Additional files. All the sequencing data generated in this study have been deposited in the Sequence Read Archive database under accession number PRJNA540186. Address is as follows: http://www.ncbi.nlm.nih.gov/sra.

## Abbreviations

TF: Transcription factor
PK: Protein kinases
QTL: Quantitative trait locus
V-H + -ATPase: Vacuolar proton pump ATPase
ROS: Reactive oxygen species
SOS: Salt overly sensitive
CDPK: Calcium-dependent protein kinase
MAPK: Mitogen-activated protein kinase
CaMK: Calcium/calmodulin-dependent protein kinase
RLK: Receptor-like kinase
DEG: Differentially expressed gene
WGCNA: Weighted gene coexpression network analysis
ASTTI: Alkaline salt tolerance trait index
RWC: Relative water content
REC: Relative electric conductivity
MDA: Malondialdehyde
GSH: Reduced glutathione
SOD: Superoxide dismutase
POD: Peroxidase
CAT: Catalase
Nr: NCBI nonredundant protein
eggnog: Evolutionary Genealogy of Genes: Nonsupervised Orthologous Groups
GO: Gene Ontology
KOG: Eukaryotic Orthologue Groups
KEGG: Kyoto Encyclopedia of Genes and Genomes
COG: Clusters of Orthologous Groups
FPKM: Fragmental per kilobase of reproduction per million mapped reads
GST: Glutathione transferase
AREB: ABA responsive element binding protein
ABF: ABRE binding factors
HSP: Heat shock protein
NGS: Next-generation sequencing
RSEM: RNA-seq by expectation maximization
