# Supplementary material for: De novo transcriptome in roots of switchgrass (Panicum virgatum L.) reveals gene expression dynamic and act network under alkaline salt stress
Source: BMC Genomics. 2021 Jan 28;22:82. doi: 10.1186/s12864-021-07368-w (PMC7841905; doi:10.1186/s12864-021-07368-w)
Supplement: Supplementary file 1 — Additional file 1: Table S1. Statistics of sample sequencing evaluation data. [file 12864_2021_7368_MOESM1_ESM.docx]

**Additional file 1: Table S1.** Statistics of sample sequencing evaluation data.

| **ID** | **Samples** | **Read Number** | **Base Number** | **GC Content** | **%≥Q30** |
| --- | --- | --- | --- | --- | --- |
| T01 | AM-0h-1 | 20,315,351 | 6,050,019,378 | 54.33% | 93.96% |
| T02 | AM-0h-2 | 21,927,466 | 6,526,725,718 | 55.51% | 93.21% |
| T03 | AM-0h-3 | 20,937,438 | 6,251,491,618 | 55.98% | 94.15% |
| T04 | AM-6h-1 | 20,995,805 | 6,266,204,308 | 55.02% | 93.60% |
| T05 | AM-6h-2 | 21,146,591 | 6,313,764,232 | 56.91% | 93.89% |
| T06 | AM-6h-3 | 22,937,276 | 6,848,872,106 | 56.16% | 93.56% |
| T07 | AM-24h-1 | 22,073,137 | 6,578,085,894 | 56.81% | 93.60% |
| T08 | AM-24h-2 | 21,408,643 | 6,395,762,320 | 57.42% | 93.61% |
| T09 | AM-24h-3 | 20,905,224 | 6,242,705,590 | 57.36% | 94.43% |
| T10 | Alamo-0h-1 | 21,351,526 | 6,356,824,352 | 55.98% | 94.61% |
| T11 | Alamo-0h-2 | 21,714,912 | 6,457,708,144 | 56.21% | 94.22% |
| T12 | Alamo-0h-3 | 20,809,433 | 6,196,668,564 | 55.80% | 94.46% |
| T13 | Alamo-6h-1 | 20,898,991 | 6,240,617,762 | 55.92% | 94.08% |
| T14 | Alamo-6h-2 | 20,487,610 | 6,100,771,616 | 56.58% | 94.59% |
| T15 | Alamo-6h-3 | 20,902,577 | 6,235,492,394 | 54.98% | 94.03% |
| T16 | Alamo-24h-1 | 20,916,069 | 6,239,739,478 | 55.67% | 94.05% |
| T17 | Alamo-24h-2 | 20,545,648 | 6,142,273,492 | 55.09% | 94.22% |
| T18 | Alamo-24h-3 | 22,050,846 | 6,590,899,218 | 56.30% | 93.57% |

AM means the genotype AM-314/MS-155, and the number behind the treated time mean three biological replicates. Read Number: total number of pair-end Reads in Clean Data; Base Number: total number of bases in Clean Data; GC Content: Clean Data GC content, which is the percentage of total bases of G and C in Clean Data; %≥Q30: The percentage of bases whose Clean Data quality value is greater than or equal to 30.
