# Supplementary material for: De novo transcriptome in roots of switchgrass (Panicum virgatum L.) reveals gene expression dynamic and act network under alkaline salt stress
Source: BMC Genomics. 2021 Jan 28;22:82. doi: 10.1186/s12864-021-07368-w (PMC7841905; doi:10.1186/s12864-021-07368-w)
Supplement: Supplementary file 2 — Additional file 2: Table S2. Statistics of reassembly results. [file 12864_2021_7368_MOESM2_ESM.docx]

**Additional file 2: Table S2.** Statistics of reassembly results.

| **Length Range (nt)** | **All Unigenes** | **AM-314/MS-155** **Unigenes** | **Alamo Unigenes** |
| --- | --- | --- | --- |
| 200-300 | 30,006(27.70%) | 19,597(26.61%) | 18,108(27.65%) |
| 300-500 | 21,049(19.43%) | 15,790(21.44%) | 13,270(20.26%) |
| 500-1000 | 22,133(20.43%) | 16,876(22.92%) | 13,835(21.12%) |
| 1000-2000 | 20,166(18.62%) | 13,493(18.32%) | 11,732(17.91%) |
| 2000+ | 14,965(13.82%) | 7,880(10.70%) | 8,547(13.05%) |
| Total Number | 108,319 | 73,636 | 65,492 |
| Total Length | 106,429,710 | 65,676,715 | 62,458,981 |
| N50 Length | 1,751 | 1,489 | 1,698 |
| Mean Length | 982.56 | 891.91 | 953.69 |

Length Range: indicates the different length intervals of Unigene; the numbers in the table indicate the number of Unigenes in the corresponding interval, the percentage in parentheses indicates the proportion of Unigene in the corresponding length interval; Total Number: indicates the total number of assembled Unigenes; Total Length: indicates the total length of assembled Unigene; N50 Length: indicates the length of Unigene's N50; Mean Length: indicates the average length of Unigene.
