# Supplementary material for: De novo transcriptome in roots of switchgrass (Panicum virgatum L.) reveals gene expression dynamic and act network under alkaline salt stress
Source: BMC Genomics. 2021 Jan 28;22:82. doi: 10.1186/s12864-021-07368-w (PMC7841905; doi:10.1186/s12864-021-07368-w)
Supplement: Supplementary file 3 — Additional file 3: Table S3. Statistics of unigene annotations. [file 12864_2021_7368_MOESM3_ESM.docx]

**Additional file 3: Table S3.** Statistics of unigene annotations

| **Annotated**  **database** |  | **All-annotated** | **Nr** | **eggNOG** | **GO** | **Pfam** | **Swiss-prot** | **KOG** | **KEGG** | **COG** |
| --- | --- | --- | --- | --- | --- | --- | --- | --- | --- | --- |
| Annotated number |  | 66,253 | 63,663 | 54,038 | 46,081 | 44,046 | 35,558 | 34,348 | 22,781 | 21,586 |
| Percentage |  |  | 96.09% | 81.56% | 69.55% | 66.48% | 53.67% | 51.84% | 34.38% | 32.58% |
