# Supplementary figures and images for: De novo transcriptome in roots of switchgrass (Panicum virgatum L.) reveals gene expression dynamic and act network under alkaline salt stress
Source: BMC Genomics. 2021 Jan 28;22:82. doi: 10.1186/s12864-021-07368-w (PMC7841905; doi:10.1186/s12864-021-07368-w)

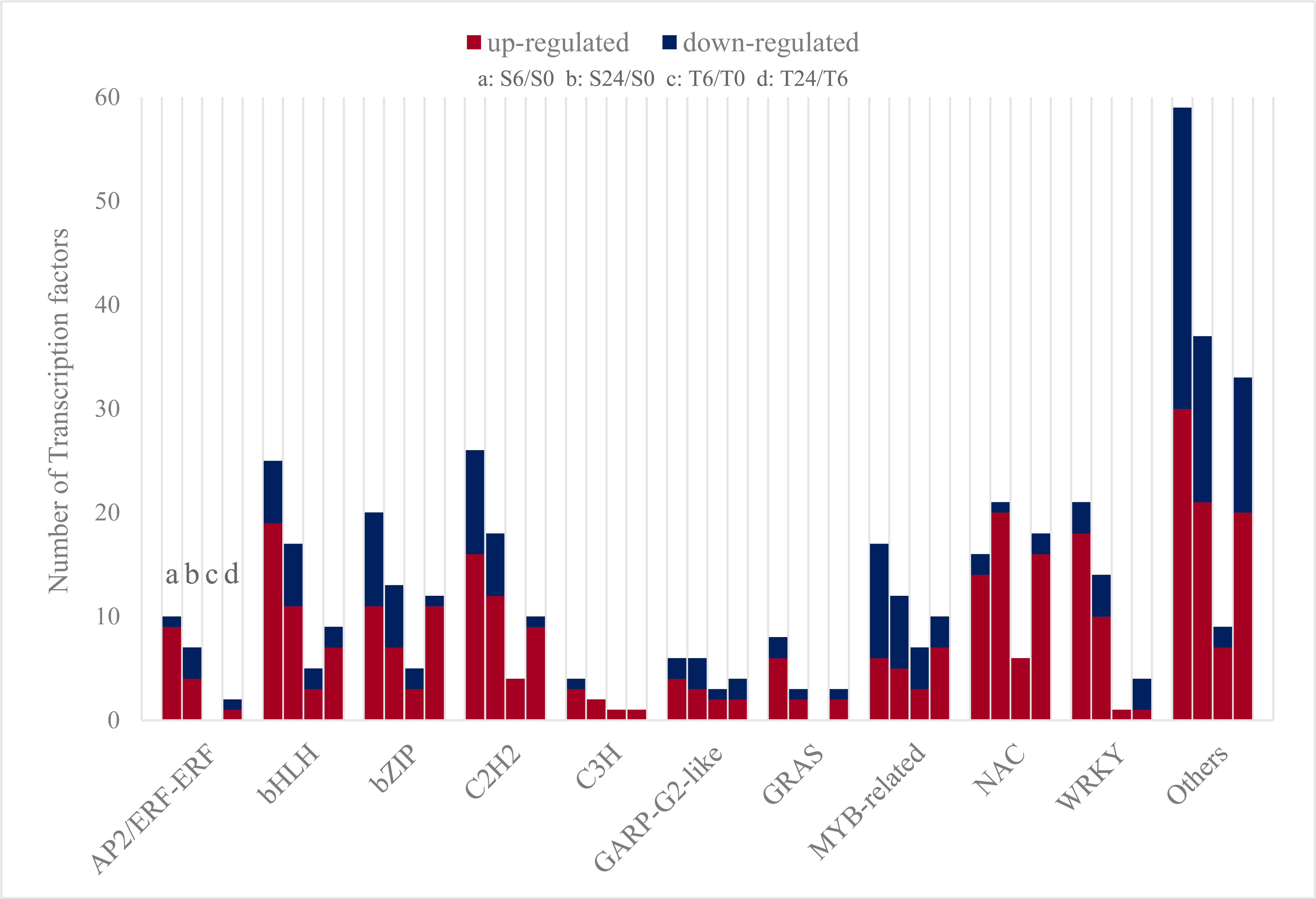

Supplement: Supplementary file 14 — Additional file 14: Figure S2. Families of differentially expressed TFs under alkaline salt stress. The X-axis represents families of TFs that were differentially expressed in the four comparisons, and the Y-axis represents the number of differentially expressed (up- or down-regulated) genes in each family. The four comparisons were S6/S0, S24/S0, T6/T0 and T24/T0, where S and T represents AM-314/MS-155 and Alamo, treated with alkaline stress treatment for either 6 or 24 h compared with control (0 h), respectively. [file 12864_2021_7368_MOESM14_ESM.tif]

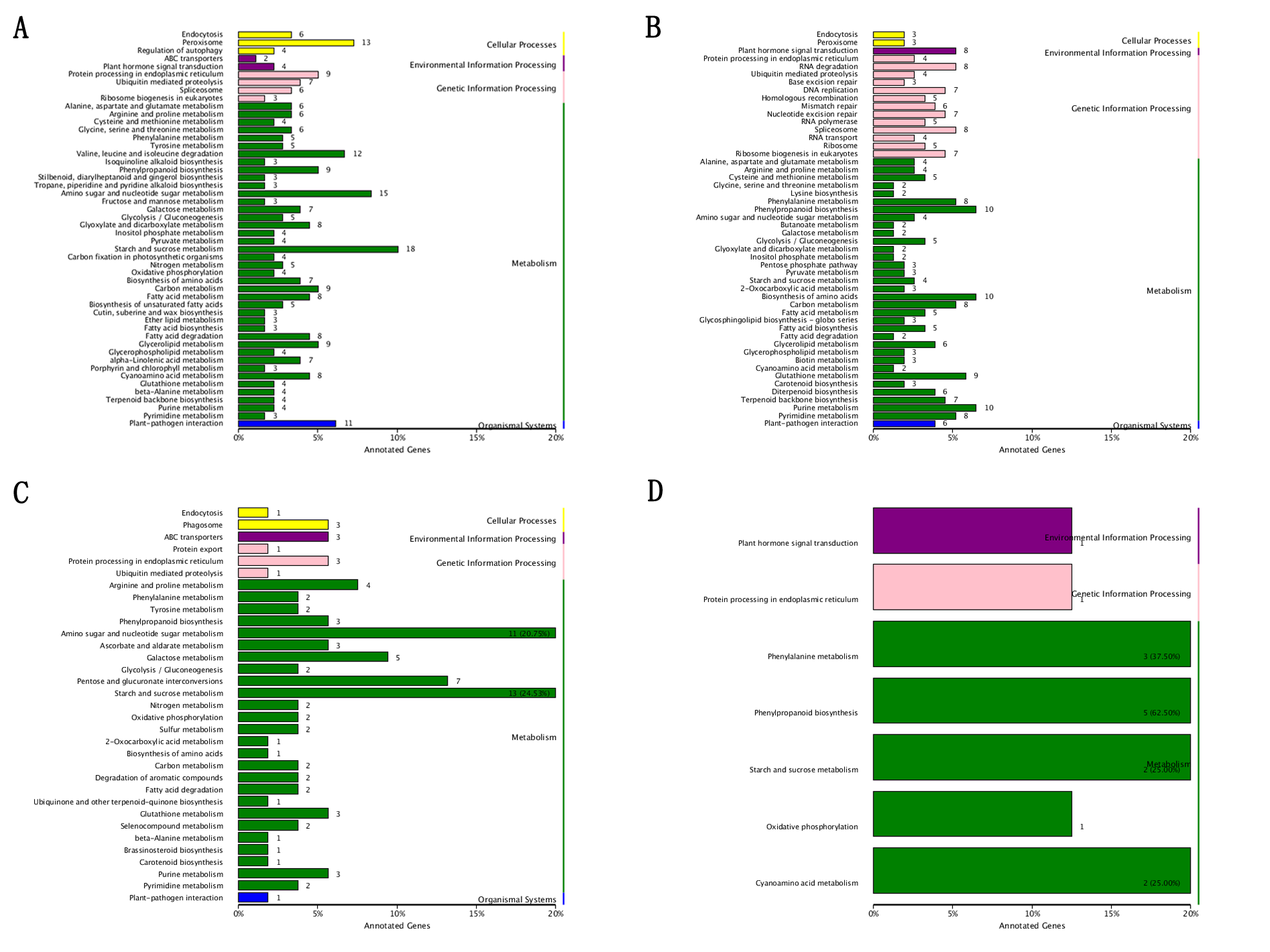

Supplement: Supplementary file 18 — Additional file 18: Figure S3. Functional enrichment analysis of modules with a higher physiological correlation. A: blue module, B: brown module, C: darkmagenta module, and D: lightsteelblue 1 module. [file 12864_2021_7368_MOESM18_ESM.tif]
