# Supplementary material for: De novo transcriptome in roots of switchgrass (Panicum virgatum L.) reveals gene expression dynamic and act network under alkaline salt stress
Source: BMC Genomics. 2021 Jan 28;22:82. doi: 10.1186/s12864-021-07368-w (PMC7841905; doi:10.1186/s12864-021-07368-w)
Supplement: Supplementary file 19 — Additional file 19: Table S16. List of primers used in qRT-PCR. [file 12864_2021_7368_MOESM19_ESM.docx]

**Additional file 19: Table S16. List of primers used in qRT-PCR**

| **Gene IDs** | **Function Annotation** | **Forward primer（5’-3’）** | **Reverse primer（5’-3’）** |
| --- | --- | --- | --- |
| CAH | PREDICTED: carbonic anhydrase, chloroplastic-like | GCTGTCAATACACTCCAGGTAG | TCCAGAGGTCGAGTCATCTT |
| DRP206 | PREDICTED: disease resistance response protein 206-like | TACGAGAAGACGAGGGACAT | CATCTTGAGGCGGAAGTAGAC |
| HPS | hypothetical protein | CATCGCAACTTCACACCATAAG | GTACATGATGCGAGTCCACTAT |
| OGH17 | putative O-glycosyl hydrolase family 17 protein | CTCCTACCTCTCCGTCAATCT | GTCTAGGAGGCTGTGGTACT |
| BG18 | PREDICTED: beta-glucosidase 18-like | ATGCCTTCTGGGTTCACTTC | GACGTTGAGCTGATGTACTCTC |
| CPRF2 | PREDICTED: light-inducible protein CPRF2-like isoform X1 | TCTTCTCCGGTCTTATACCTCTAC | TTCTCCAACAACCACGTACTC |
| GST23 | PREDICTED: glutathione transferase GST 23-like | ACTTGTCGGCGATGAACTGG | TCTGCGAGTCCCTCGTCAT |
| CUF15 | PREDICTED: cytosolic sulfotransferase 15-like | TGCGCTACCTCCATCATTTAC | GACTGGAAGGCTCACATCAC |
| PDH2 | PREDICTED: proline dehydrogenase 2, mitochondrial-like isoform X2 | GTCGATCCATCCTCCATCTTG | GAGGTGGTGGTGATGTTAGTT |
| MTN5 | PREDICTED: metal transporter Nramp5-like | CCGGAGTAAGTGCCTGTTATG | CTCGACTCCTCCTCCTTTCT |
| CYP-5 |  | CACTACAAGGGAAGCACATTCCA | TTCACCACCCCTTCCATCAC |
